# Transcriptomic and Proteomic Analyses of Celery Cytoplasmic Male Sterile Line and Its Maintainer Line

**DOI:** 10.3390/ijms24044194

**Published:** 2023-02-20

**Authors:** Haoran Wang, Qing Cheng, Ziqi Zhai, Xiangyun Cui, Mingxuan Li, Ruiquan Ye, Liang Sun, Huolin Shen

**Affiliations:** 1 Beijing Key Laboratory of Growth and Developmental Regulation for Protected Vegetable Crops, China Agricultural University, Beijing 100193, China; 2 Department of Vegetable Science, College of Horticulture, China Agricultural University, No. 2 Yuanmingyuan Xi Lu, Haidian District, Beijing 100193, China

**Keywords:** celery, proteomic analysis, transcriptomic analysis, cytoplasmic male sterility

## Abstract

Male sterility is a common phenomenon in the plant kingdom and based on the organelles harboring the male-sterility genes, it can be classified into the genic male sterility (GMS) and the cytoplasmic male sterility (CMS). In every generation, CMS can generate 100% male-sterile population, which is very important for the breeders to take advantage of the heterosis and for the seed producers to guarantee the seed purity. Celery is a cross-pollinated plant with the compound umbel type of inflorescence which carries hundreds of small flowers. These characteristics make CMS the only option to produce the commercial hybrid celery seeds. In this study, transcriptomic and proteomic analyses were performed to identify genes and proteins that are associated with celery CMS. A total of 1255 differentially expressed genes (DEGs) and 89 differentially expressed proteins (DEPs) were identified between the CMS and its maintainer line, then 25 genes were found to differentially expressed at both the transcript and protein levels. Ten DEGs involved in the fleece layer and outer pollen wall development were identified by Gene Ontology (GO) and Kyoto Encyclopedia of Genes and Genomes (KEGG) analyses, most of which were down-regulated in the sterile line W99A. These DEGs and DEPs were mainly enriched in the pathways of “phenylpropanoid/sporopollenin synthesis/metabolism”, “energy metabolism”, “redox enzyme activity” and “redox processes”. Results obtained in this study laid a foundation for the future investigation of mechanisms of pollen development as well as the reasons for the CMS in celery.

## 1. Introduction

Celery (*Apium graveolens* L., 2n = 2× = 22), an annual or biennial herbage species that belongs to the Apiaceae family, is one of the most popular vegetables worldwide [1]. Celery has an umbel inflorescence which consists of hundreds of tiny flowers, and an inconsistent maturation time of the stamens and pistils results in extreme difficulty in hand pollination and the application of heterosis. Therefore, in order to take advantage of the heterosis in celery, male sterility must be utilized in the production of hybrid seeds. So far, two types of male sterility have been discovered in celery, genic male sterility (GMS) and cytoplasmic male sterility (CMS) [2]. Although GMS is stable and the fertility-restoring gene is easier to find, the elimination of the 50% of fertile offspring limits its applications. In contrast, due to the matrilineal inheritance, the offspring the CMS line is male sterile [3]. In higher plants, CMS is mainly caused by the rearrangement of mitochondrial DNA, which results in the inability to produce pollen or the production of abnormal pollen [4,5]. CMS has been applied in the production of hybrid seeds in many crops, which could effectively decrease the cost as well as improve the purity of the hybrid seeds [3].

In flowering plants, the development of pollen from sporogonia to mature pollen consists of a series of complex events regulated by multiple genes [6,7], and any obstacle to the anther development may lead to pollen abortion and finally result in male sterility [8]. So far, two hypothesized mechanisms for the CMS have been proposed: (I) CMS inhibits energy production by destroying mitochondrial electron transport chain complexes; and (II) CMS damages the normal growth of cells [9,10]. For verifying the above-mentioned hypothesis, many efforts have been made at different levels. With the development of the high-throughput analytical approaches, the transcriptomic, proteomic and metabonomic bases of CMS have been gradually revealed [1]. Differentially expressed genes (DEGs) involved in protein synthesis and metabolism were identified in male sterile and maintainer lines of *pol* CMS and SaNa-1A CMS in *Brassica napus* [11,12]. Transcriptomic, cytological and biochemical analyses of *Gossypium hirsutum* showed that the ability of male sterile lines to scavenge reactive oxygen species (ROS) is decreased, and the ROS released by mitochondria, as a signal molecule in the nucleus, triggered the formation of abnormal tapetum [13,14]. Moreover, proteomic analysis demonstrated that down-regulation of proteins related to carbohydrate and energy metabolism, photosynthesis and flavonoid synthesis in the anthers of the CMS *B. napus*, indicative of their critical roles in pollen development [15]. In cabbage, the transcriptomic and proteomic analyses revealed gibberellin and sporopollen syntheses as important pathways involved in the formation of CMS [16]. Integrated analysis of the transcriptome and proteome provided an overview of complete regulatory network of CMS in Chinese cabbage [16], *Brassica napus* [17], pepper [18], and pigeon pea [19]. In cotton, 550 differentially expressed transcript-derived fragments (TDFs) and at least 1013 proteins were detected in anthers at various developmental stages, and UDP-glucuronosyl/UDP-glucosyltransferase, 60S ribosomal protein L13a-4-like, glutathione S-transferase, heat shock protein Hsp20, ATPase, F0 complex, and subunit D were considered to be involved in the regulation of the CMS of Yamian A [20].

With the development of high-throughput technologies, more and more studies use transcriptomic and proteomic sequencing to identify the candidates’ underlying male sterility. Recently, a new strategy named data-independent acquisition (DIA) has been used to analyze protein abundance across cotton CMS lines, and DEPs participating in oxidoreductase activity, carbohydrate metabolism, fatty acid metabolism, cell aging, wax or cutin deposition and signal transduction have been identified [21]. Later, by using virus-induced gene silencing (VIGS) system, *Gh_A11G1250*, which is involved in ROS response, has been confirmed to play a part in the anthers abortion [21]. In another aspect, iTRAQ and PRM-based assay have been applied in the identification of candidate genes underlying temperature-sensitive CMS in *Aegilops kotschyi*, and *TaEXPB5* has been screened out. Furthermore, silencing of *TaEXPB5* resulted in pollen abortion and declined fertility [22]. In *G. hirsutum*, transcriptome analysis of GMS mutant and its background line discovered a significant DEG, *GhGLP4*, of which the down-regulation resulted in pollen sac closure, stigma exertion, filament shortening and complete male sterility as well as the expression differences in some oxidase genes [23].

In regard to celery, so far there are only few studies focusing on the CMS, of which most have focused on the genetical, histological, physiological and biochemical aspects, but few puts effort into the illustration of the mechanism of CMS at the transcriptional and proteomic levels. In this study, the CMS line W99A and its maintainer line W99B were employed, and flower buds at the tetrad stage were selected for transcriptome and proteome analyses to identify the differential genes/proteins between them. Among those genes or proteins, twenty-five genes were differentially expressed at the transcript and protein levels, which were mainly enriched in the pathways of “phenylpropanoid/sporopollenin synthesis/metabolism”, “energy metabolism”, “redox enzyme activity” and “redox processes”. Ten differentially expressed genes (DEGs) involved in the fleece layer and outer pollen wall development were identified. Later, the potential relationship of the above-mentioned genes or proteins with the formation of CMS in celery has also been explored. Results obtained in this study are valuable for subsequent studies as well as the breeding of celery varieties.

## 2. Results

### 2.1. Morphology of the Sterile and Fertile Flowers

**Figure 1:**
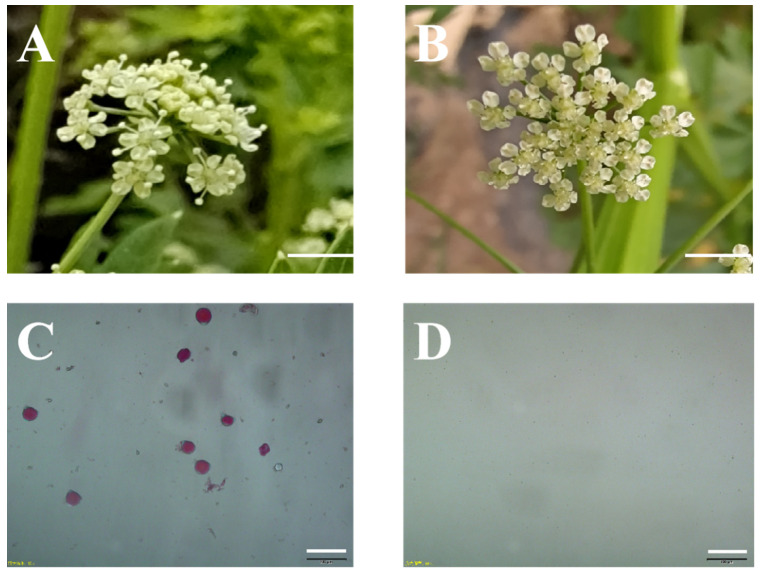
Anthesis flowers and pollen staining of the CMS and maintainer lines. (**A**) Flower of maintainer line W99B, scale = 4 cm; (**B**) flowers of sterile line W99A, scale = 4 cm; (**C**,**D**) pollen staining using Alexander’s dye, scale = 100 µm.

Morphological investigation was performed on celery male sterile line W99A and its maintainer line W99B, but no distinct difference was observed in the vegetative organs between the two lines. However, the anther structure of the CMS lines was obviously different from that of the maintainer line. Particularly, in the anthesis flowers of the maintainer line the dehiscent anthers were plump and raised up by the elongated filaments, and pollens can be easily seen on the surface of the anthers. In contrast, in anthesis flowers of the CMS line, the filaments were short, and the anthers were shriveled and indehiscent, on the surface of which pollens could not be seen (Figure 1A,B). Pollen staining showed that pollens from the maintainer line were well developed and plump, with 100% vitality, whereas no pollen could be collected from the CMS line (Figure 1C,D).

### 2.2. Overview of the RNA-Seq Data

Transcriptomes of flower buds of CMS line W99A and its maintainer line W99B were analyzed by RNA-seq. Three biological repeats were collected: T01, T02, and T03 for W99A, and T04, T05, and T06 for W99B. For each sample, 83.90–85.30% of the clean reads were mapped to the celery genome and a total of 36,146,434, 37,252,942, 39,789,111, 37,027,177, 36,598,988 and 44,905,793 unique reads were identified in T01 to T06, respectively (Table S1). The correlation coefficients (R^2^) among different repeats in W99A were 0.977 (T01 and T02), 0.982 (T01 and T03) and 0.971 (T02 and T03), and in W99B were 0.868 (T04 and T05), 0.870 (T04 and T06) and 0.966 (T05 and T06) (Figure S1). Pairwise comparison analyses revealed that in each line the similarities between the samples were less than the similarities within the samples, indicating the RNA-seq data met the requirements of the subsequent analyses.

**Figure 2:**
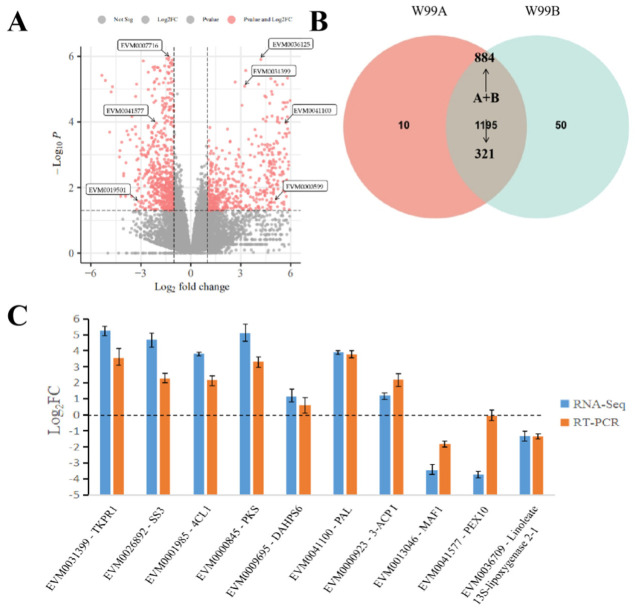
Differentially expressed gene profiles obtained from the transcriptomic analyses. (**A**) Volcano map of up- and down-regulated genes between the male sterile W99A and the male fertile line W99B; (**B**) comparison of differentially expressed genes (DEGs) identified in the sterile W99A and the fertile line W99B; (**C**) DEGs selected from transcriptome was verified by RT-PCR, and the material was W99A. Buds in tetrad stage were selected, and each sample was repeated three times.

A total of 1255 differentially expressed genes (DEGs) were identified (Figure 2A), which met the following requirements: (i) the absolute value of a gene’s log2Fold change (W99A/W99B) was greater than or equal to 1; (ii) the false discovery rate (FDR) of a gene was less than or equal to 0.01. Among those DEGs, 403 and 582 were down- and up-regulated in W99A, respectively (Table S2; Figure 2A). Additionally, 10 and 50 genes were specifically expressed in W99A and W99B, respectively (Table S2; Figure 2B). When considering the physiological positions of the DEGs in celery genome, chr3 (121), chr5 (118) and chr2 (111) had the highest DEG number among all the 11 chromosomes (Figure S2).

### 2.3. GO and KEGG Analyses

To gain insight into the transcriptome changes, the differentially expressed genes were annotated in the database, and their functional distribution was detected. Gene ontology (GO) analysis was conducted. In the “molecular function” category, the DEGs were mainly enriched in the terms of “integral component of the membrane” and “endoplasmic reticulum lumen”; in the category of “biological processes”, the DEGs were mainly enriched in the terms of “sporopollenin biosynthesis process” and “regulation of ARF protein signaling”; as to the “molecular functional” category, the major enriched terms were “oxidoreductase activity” and “pseudouridine synthase activity” (Figure S3A). Among the above-mentioned genes, DEGs enriched in “sporopollenin biosynthesis process” and “oxidoreductase activity” were all up-regulated in the maintainer lines W99B; meanwhile, in the term of “membrane components”, 82% of the DEGs were down-regulated in the sterile lines W99B (Table S3).

After observing the functional distribution of DEGs, in order to further understand their causal relationship and specific functions KEGG analysis method was adopted to analyze their enrichment pathways. In the KEGG analysis, 260 DEGs were successfully classified into different pathways, and the most enriched pathways were sucrose metabolism (27 DEGs, 10.38%), plant–pathogen interactions (24 DEGs, 9.23%) and phenylpropanoid biosynthesis (23 DEGs, 8.85%) (Figure S3B). Among the 20 most significantly enriched KEGG pathways, “phenylpropane metabolism”, “keratin, lipid and wax biosynthesis”, “phenylpropane biosynthesis”, “starch and sucrose biosynthesis”, “phenyl propane biosynthesis” and “starch and sucrose metabolism” were involved in the regulation of pollen development (Table S4). In the first two pathways, expression of 16 DEGs was decreased in the CMS W99A line. Meanwhile, more than 85% of DEGs in the “phenylpropane biosynthesis” and “starch and sucrose biosynthesis” pathways were down-regulated in the W99A lines.

**Figure 3:**
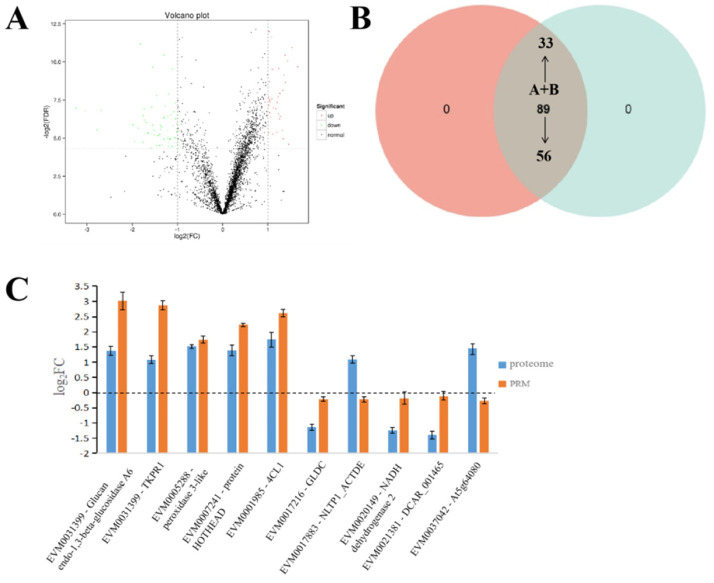
Differentially expressed protein profiles obtained from the proteomic analyses. (**A**) Volcano map of up- and down-regulated proteins between the male sterile W99A and the male fertile line W99B; (**B**) comparison of differentially expressed proteins (DEPs) identified in the sterile W99A and the fertile line W99B; (**C**) DEPs selected from proteomics was verified by PRM, and the material was W99B. Buds in tetrad stage were selected, and each sample was repeated three times.

To verify the expression variations in the DEGs, the expression levels of ten pollen develop-related DEGs (Table S5), including two tapetum formation-related genes (*EVM0001985* and *EVM0000845*) and eight pollen exine formation-related genes, were investigated by RT-PCR in which the materials were as same as what has been analyzed in the RNA-seq. As shown in Figure 3, expression patterns of the ten tested genes were consistent with those observed in RNA-seq, indicating the RNA-seq data were reliable.

### 2.4. Differentially Expressed Proteins between W99A and W99B

To identify the proteins that were involved in the regulation of CMS in celery, proteomic analysis was performed with the same samples in RNA-seq analysis. Differentially expressed proteins should meet the following requirements: (i) the difference multiplier FC ≥ 2; and (ii) *p*-value ≤ 0.05 in the *t*-test (Figure 2C). A total of 89 differentially expressed proteins (DEPs) were screened out, of which 56 and 33 were down- and up-accumulated in the CMS W99A line, respectively (Table S6; Figure 3A).

### 2.5. Functional Classification of DEPs

GO analysis was used to classify the DEPs according to their functions, and the DEPs were discovered to be classified into 26 functional groups. Among those groups, 12 belonged to the biological process (the lowest category was “metabolic process”), 8 belonged to the cellular components (the lowest category was “cell part”), and 6 belonged to the molecular functions (the lowest category was “catalytic activity”) (Figure S4A). The top three terms that had the highest number of DEPs were “extracellular region” (cell composition), “lipid transport” (biological processes), and “lipid binding” (molecular functions). The above-mentioned terms and their proteins such as “lipid transport and binding” and “oxidoreductase activity” were all down-regulated in male sterile lines, indicating that they may be, at least directly or indirectly, related to the production of celery CMS (Table S7).

**Figure 4:**
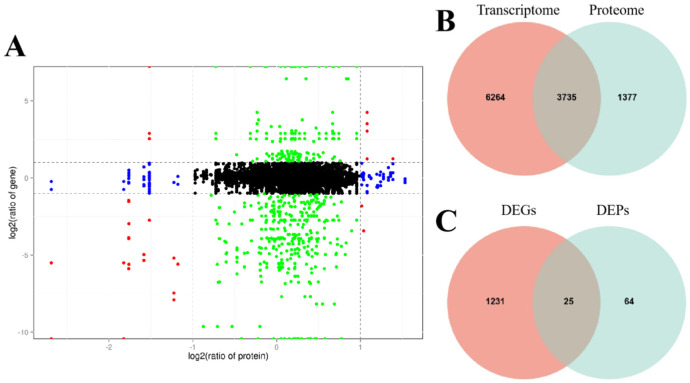
Differentially expressed gene/protein profiles obtained from transcriptomic and proteomic analyses. (**A**) Scatterplots of the relationship between genes quantified in both transcriptome and proteome analysis; (**B**) Comparison of all the proteins and genes identified in the sterile W99A and the fertile line W99B; (**C**) Comparison of all the DEGs and DEPs identified in the sterile W99A and the fertile line W99B. Quadrants 1 and 9 indicate opposite protein and mRNA expression trends; quadrants 3 and 7 indicate the same protein and mRNA expression trends; quadrants 2 and 8 indicate no change in protein and differential mRNA expression; quadrants 4 and 6 indicate no change in mRNA and differential protein expression. Orange circle represents DEGs detected by transcriptomics, and green circle represents DEPs detected by protein omics.

KEGG analysis revealed that those DEPs were mainly enriched in metabolism and genetic information processing pathways. The metabolism pathway was the most common category in all the datasets, which mainly consisted of glyoxylate and dicarboxylate metabolism, carbon metabolism terms and metabolism of various amino acids (Figure S4B). In the aspect of expression patterns, the DEPs belonging to the glutamic acid (proline precursor) carbon fixation during photosynthesis, glyoxylate and dicarboxylate metabolism were up-accumulated in the CMS line. In contrast, alienation-related DEPs involved in the starch and sucrose metabolism, fructose and mannose metabolism were down-accumulated (Figure 4C). Additionally, DEPs related to pollen development, such as proteins involved in phenylpropanoid synthesis (the precursor of pollen wall) and phenylpropane metabolism (whose related metabolites maintain cell wall stability), were down-accumulated in the male sterile lines (Table S8).

In order to verify the accuracy of the iTRAQ, 10 fertility-related DEPs were selected for targeted parallel reaction monitoring (PRM) assay and 8 showed good consistencies with iTRAQ data. Among the 8 DEPs, three were up-regulated (*EVM0017216*, *EVM0020149* and *EVM0021381*) and five were down-regulated (*EVM0037081*, *EVM0031399*, *EVM0005288*, *EVM0007341* and *EVM0001985*) in the male sterile line (Figure 3C).

### 2.6. Transcriptome and Proteome Conjoint Analysis

**Figure 5:**
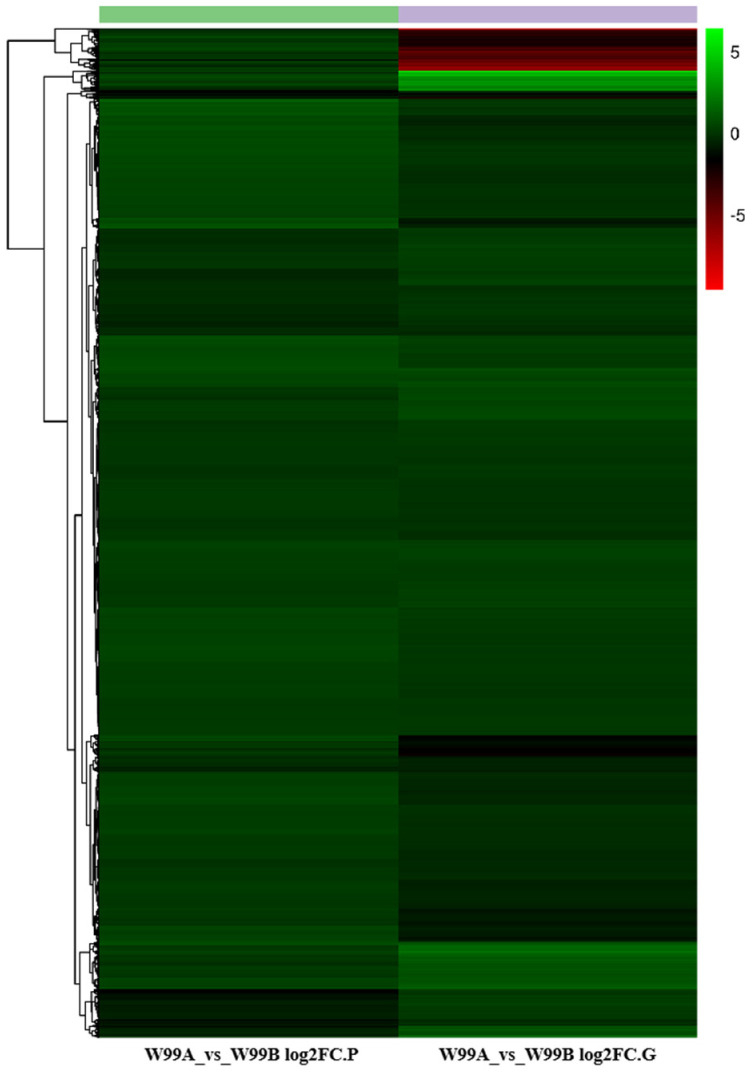
Clustering maps of proteomic and transcriptomic expression patterns. The columns of the graph represent differential groupings of proteins or genes (proteins with .P suffix on the left and genes with .G suffix on the right), and the rows represent log2FC values of proteins or genes, where genes and proteins in the same row of the same differential grouping are correlated. See Table S12 for the expression of DEGs/DEPs in different omics in cluster analysis.

For further understanding about the molecular mechanism of CMS in celery, transcriptome and proteome conjoint analysis was conducted and 3735 genes were detected in both the transcriptomic and proteomic assays, of which 1344 were differentially expressed (Figure 4A). Among the 1255 genes, 64 genes were only detected to be differentially accumulated in the proteomic data (Figure 4B), and meanwhile, 1230 genes were only differentially expressed in the transcriptomic data, and only 25 genes were observed to be differentially expressed in both of the omics datasets (Figure 4C and Figure 5, Table S9).

**Figure 6:**
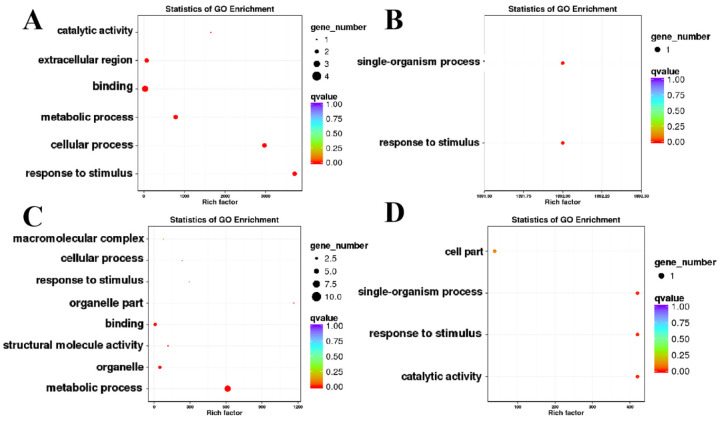
Gene ontology (GO) enrichment analysis of co-DEGs-DEPs genes. (**A**) GO annotation results for genes with opposite protein and mRNA expression trends; (**B**) GO annotation results for proteins with opposite protein and mRNA expression trends; (**C**) GO annotation results for genes with identical protein and mRNA expression trends; (**D**) GO annotation results for proteins with the same protein and mRNA expression trends. In the figure, each circle represents a GOTerm, the ordinate represents the name of GO, and the abscissa represents the Enrichment Factor. The greater the enrichment factor, the more significant the enrichment level of differentially expressed genes in this pathway. The color of the circle represents qvalue, and qvalue is Pvalue after multiple hypothesis testing and correction. The smaller the qvalue is, the more reliable the enrichment significance of differentially expressed genes or proteins in this pathway is. The size of the circle indicates the number of genes or proteins enriched in the pathway. The larger the circle, the more genes there are.

**Figure 7:**
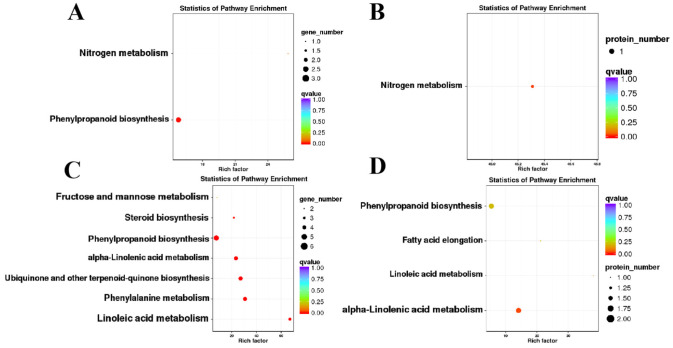
Kyoto Encyclopedia of Genes and Genomes (KEGG) enrichment analysis of co-DEGs-DEPs genes. (**A**) GO annotation results for genes with opposite protein and mRNA expression trends; (**B**) KEGG enrichment information for proteins with opposite trends in protein and mRNA expression; (**C**) KEGG enrichment information of genes with the same protein and mRNA expression trends; (**D**) KEGG enrichment information for proteins with the same trend of protein and mRNA expression.

Since the 25 genes were common to two omics, GO and KEGG analyses were performed. Among the 25 genes, six showed opposite expression trends between the transcriptomic and proteomic datasets, which were mainly enriched in the terms of “response to stimulus”, “cellular process”, “metabolic process”, “catalytic activity”, “binding” and “extracellular region” (Figure 6A,B and Figure S5A,B). In another aspect, the remaining 19 genes, which showed consistent expression patterns between the two omics, were enriched in the terms of “metabolic process”, “organelle”, “structural molecule” and “catalytic activity” (Figure 6C,D and Figure S5C,D). Among the 19 genes, 15 and 4 genes were down- and up-regulated in CMS line, respectively (Table S10). Similar to what was observed in the GO analysis, in the KEGG analysis DEPs were enriched in the “phenylpropanoid biosynthesis”, “nitrogen metabolism”, “alpha-linolenic acid metabolism” and “phenylpropanoid biosynthesis” pathways (Figure 7A–D and Figure S6A–D, Table S11).

In addition to what is mentioned above, four of the 25 genes, including *EVM0031399*, *EVM0026892*, *EVM0001985* and *EVM0041100*, were found to be the homologs of Arabidopsis *LOC108217645* (*TKPR1*), *LOC108198869* (*STR*), *LOC108198861* (*4CL*) and *LOC108224761* (*PAL*), respectively. *TKPR1*, *STR*, *4CL* and *PAL* are all involved in the pollen development, suggesting their homologs in celery may also play the similar roles. Since all the genes were down-regulated in the CMS W99A line, it can be presumed that they may be involved in the regulation of CMS in celery.

### 2.7. Determination of the Peroxides and Activities of Peroxides Eliminating Enzymes

**Figure 8:**
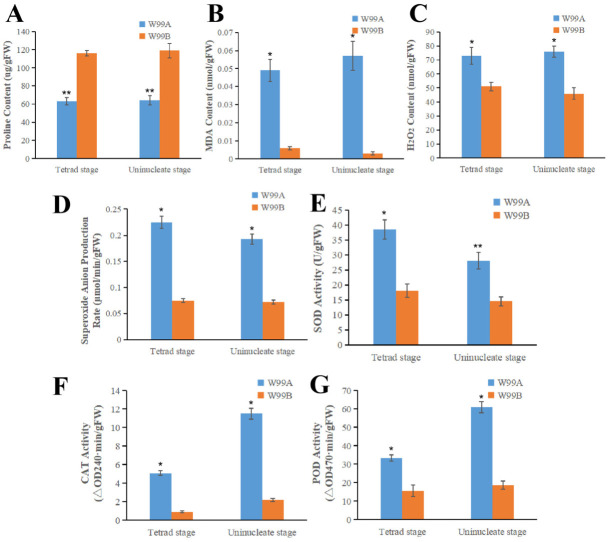
Detection and analysis results of biochemical indexes of W99A and W99B. (**A**–**D**) show proline content, MDA content, hydrogen peroxide content and the rate of superoxide anion production. (**E**–**G**) show SOD activity, CAT activity, and POD activity, respectively. “*” and “**” represent significant levels of difference at *p* < 0.05 and *p* < 0.01, respectively. Mean ± standard deviation, repeated three times.

Since DEGs and DEPs were enriched in “oxidoreductase activity” and “redox process” terms, contents of proline, malondialdehyde (MDA), hydrogen peroxide, activities of superoxide dismutase (SOD), catalase (CAT) and peroxidase (POD) as well as the superoxide anion production rate were measured in the flower buds at tetrad and uninucleate stages. The proline content and CAT activity of the CMS line were, respectively, 56% and 45% of those of the maintainer line (Figure 8A,F). In contrast, the activities of SOD and POD in the CMS line were, respectively, 2.18 and 2.78 times as many as those in the maintainer line (Figure 8E,G). Similarly, the contents of MDA and hydrogen peroxide in the CMS line were 9.24 and 1.55 times as many as those in the maintainer line, respectively (Figure 8B,C). The MDA content was influenced by the peroxidation of the cytomembrane, which was mainly caused by the excessive accumulation of the ROS. As to the production rate of superoxide anion in the CMS line, it was 1.68 times as much as that in the maintainer line (Figure 8C).

### 2.8. Comparative Study of the Expression of Fertility-Related Genes between W99A and W99B

**Figure 9:**
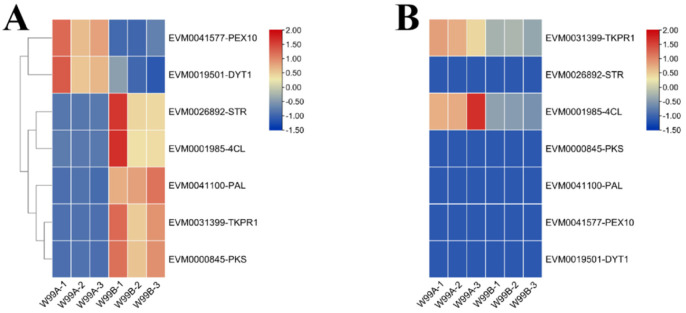
Genes involved in pollen development in DEGs and their expression analysis in protein omics. (**A**) shows the expression of selected related genes in W99A and W99B transcriptome data, followed by “-” and designation of homologous gene in Arabidopsis after sequence alignment; (**B**) shows the expression of these genes in protein group.

Some genes related to the anther/pollen development of Arabidopsis were compared in the celery genome to observe their expression trend in each group. The transcriptomic and proteomics are shown in Figure 9. Only two genes were up-regulated in male sterile lines, namely, *EVM0041577*-*PEX10*, which regulates peroxidase synthesis, and *EVM0019501*-*DYT1*, which regulates anther tapetum development; whereas genes involved in regulating plant pollen development (*EVM0031399*-*TKPR1*, *EVM0026892-STR*, *EVM0001985-4CL* and *EVM0041100-PAL*) and polyketones (phenols, stilbenes) (*EVM0000845-PKS*) were down-regulated in male sterile lines. These genes may be involved in the process of male sterility in celery in different ways.

## 3. Discussion

Male sterility is the result of the variations at the transcript and protein levels, which are caused by the mutations of the stamen and pollen development-related genes [2]. Therefore, analysis of the transcriptomic and proteomic changes is not only very important for deepening our understanding about the mechanism of male sterility, but also valuable for the application of male sterility in breeding. As to celery, little has been known about the mechanism underlying CMS. Thus, in this study, comparative analyses have been performed between the CMS and the fertile lines at the transcriptomic and proteomic levels.

In the previous histological investigations in our laboratory the male sterility in W99A occurred at the tetrad stage, and based on this, anthers bearing microspores at the tetrad stage were collected and the transcriptome and proteome were sequenced. The results showed that the differentially expressed genes/proteins were mainly enriched in some pathways involved in pollen development. The genes/proteins involved in the discussion are described in Table S13.

The main function of the sporopollenin is to form pollen exine and provides protection against environmental stress. Mutants with defective pollen wall structure usually show decreased fertility or complete loss of fertility [24,25]. In the biosynthesis of sporopollenin, fatty acids need to be transported into the endoplasmic reticulum by a series of enzymes and then transferred into sporopollenin precursors after hydroxylation and acylation [26]. Therefore, whether sporopollenin synthesis or the function of endoplasmic reticulum is disturbed, the fertility of anthers would be damaged directly or indirectly and eventually lead to male sterility. In Arabidopsis, the fatty acids are catalyzed by ACOS5, a member of the 4CL family, to form fatty acyl CoAs, which is the substrate of sporopollenin [27]. Then, triketone or tetraketo α-pyranone is synthesized from fatty acyl CoAs and malonyl coenzyme A, which is catalyzed by PKS, a member of the PKS III superfamily that accumulated in the tapetum [28]. Later, triketone or tetraketo α-pyranone is transferred into the sporopollenin intermediate products [29], which are later restored by *TKPR1* and *TKPR2* [30]. Genetic and biochemical studies showed that *ACOS5*, *PKS* and *TKPR1* are involved in the formation of sporopollen metabolites in the tapetum of *Arabidopsis*, which promotes the formation of pollen wall [31]. A similar pathway was also found in tobacco [32], moss [33], Hypericum trees [34] and Canola [35] and other plants, indicating its importance in the pollen development in plant. In citrus, reduced expression of *4CL* led to male sterility [36], and mutants of *acos5* and *acos12* in both rice and *Arabidopsis* resulted in the abnormal development of pollen wall as well as different degrees of the reduced fertility [37]. Similarly, knockout of *OsPKS1* in rice exhibited complete male sterility [38], and meanwhile point mutations in *OsPKS2* affected pollen wall formation and eventually led to male sterility [39]. In rice, loss-of-function mutation of *OsTKPR1* caused the delay of tapetum degradation, reduction in the levels of anther cuticular lipids, impaired Ubisch body and pollen exine formation, and finally resulted in the complete male sterility [40]. In contrast, the *tkpr2* mutant in *Arabidopsis* only resulted in the decline of fertility, which could be attributed to the damaged pollen outer wall [30]. Although the expression of mRNA and protein is not consistent in our research, considering that the process from mRNA to protein is not direct, it is normal that the time factor will lead to inconsistent abundance of both in the same sample. From what is mentioned above, it can be presumed that the down-regulation of the expression of the above three celery genes in W99A may lead to impaired pollen outer wall and consequently pollen abortion.

In addition to the above-mentioned pathways, results in this experiment also revealed that the “starch and sucrose metabolism” and “glycolysis/glycogenesis” pathways were also altered in W99A. These two pathways are essential for the energy production in organisms, of which the abnormalities may interfere with the energy metabolic processes in mitochondria and finally result in male sterility [41]. In pepper, the contents of ATP and NADH significantly decreased in the maintainer line during the abortion stage of pollen [42]. In Honglian CMS rice, the abnormal expression of *atp6-orh79* led to the decreased ATPase activity, which further caused pollen abortion [43]. In addition, related studies in tobacco and *B. napus* have demonstrated that the ATP content significantly decreased in male sterile lines [44,45]. In soybean CMS sterile line NJCMS1A, eight DEGs in the glycolytic/gluconeogenic pathway and four DEGs in the starch and sucrose metabolic pathways were all down-regulated [46]. Consistently, in the proteomic analysis of wheat P-type CMS, accumulation of 34 proteins in the starch and sucrose metabolism pathways was also impacted which was thought to be one of major reasons for the male sterility [47]. Therefore, it can be speculated that the male sterility of W99A is related to the changes of these two pathways. In those two pathways, two interested genes were identified, including *EVM0000599* and *EVM0014167*. *EVM0000599* is a homolog of Arabidopsis *ALDH3H1* gene, which belongs to the aldehyde dehydrogenase family. A member of this family, *Rf2a*, has been confirmed to be a recovery gene of maize T-type CMS [48]. Moreover, two aldehyde dehydrogenase genes, *ALDH2a* and *ALDH2b*, which highly expressed in rice panicles, were considered to be involved in the regulation of fertility [49]. *EVM0014167* encodes a homolog of Arabidopsis *ALDH2B4* gene and a maze *ALDH2B4* gene has been discovered to be required for anther development [50]. Numerous studies have shown that carbohydrates such as sucrose not only provide nutrients for anther development, but also affect pollen and anther development as a signal [51]. In cucumber, mutation of a sucrose transporter gene, *CsSUT1*, caused male sterility through altering the carbohydrate supply [52]. Meanwhile, interfering with sugar metabolism and translocation in anthers also obviously damaged the development of rice pollen and eventually led to male sterility [53]. Thus, the down-regulation of the expression of *EVM0000599* and *EVM0014167* could be one of reasons for the celery male sterility.

Mutations in the “oxidoreductase activity” pathway also impact anther and pollen development and result in male sterility. Oxidoreductase plays an important role in the homeostasis of ROS in plants. Therefore, inhibition of the oxidoreductases could cause a large amount of ROS accumulation [54]. PCD in the chorioallantoic layer is an important step in pollen development, and abnormal ROS levels promote the occurrence of abnormal PCD in plants, which leads to male sterility [55]. In *WA352*, the rice chorionic felt layer can be triggered prematurely by PCD, leading to pollen abortion [56]. Premature or delayed PCD of the chorionic felt layer in Arabidopsis all led to the occurrence of male sterility [57]. Similarly, some studies have reported that the adenine phosphoribosyl transferase activity deficient mutants in *Arabidopsis* were male sterile, which could be attributed to the abortion of pollen [58]. Additionally, studies in *Arabidopsis* and rice have demonstrated that disruption of the genes encoding UGPase or GSL altered callose metabolism, and later led to the defective microsporogenesis, which ultimately resulted in male sterility [59,60]. In this experiment, we measured the contents of the substances that were related to oxidoreductase activity and oxidoreductase process, in celery male sterile line W99A and maintainer line W99B. The results showed that the content of proline in celery was consistent with that in wheat [61], and inhibition of the proline synthesis was able to induce the male sterility [62]. In contrast, activities of SOD and POD, as well as the contents of MDA and hydrogen, were higher in the sterile lines than in the keeper lines. In wheat Aegilops uniaristata CMS line, it was found that the activities of SOD and POD in male sterile anthers were higher than those in the fertile anthers in the whole process of anther development, which may prevent the excessive accumulation of ROS [63]. Similar results were obtained in the study of rice CMS, presumably caused by fluctuations in MDA content [64]. Similarly, in both cotton [65] and kenaf [66], the MDA content of male sterile lines was significantly higher than that of the maintainer lines. The excessive accumulation of O^2-^ and H_2_O_2_ found in CMS lines of wheat may be attributed to the changes of expression levels of the genes encoding antioxidant enzymes [67]. The results mentioned above are consistent with those obtained in this experiment, indicating that the abnormal expression of genes/proteins involved in the regulation of the activity of oxidoreductase and redox process in celery male sterile line W99A may be one of the major reasons for the emergence of celery CMS. Therefore, it can be speculated that the redox pathway is also involved in the fertility changes of CMS. Compared with that of W99B, expression of the genes in the “oxidoreductase activity” pathway in W99A showed decreasing trends. Among those genes, *EVM0031399* and *EVM0041100*, whose homologs in *Arabidopsis* are *TKPR1* and *PAL*, respectively, attracted our attention. In previous studies, defective pollen wall has been observed in the plants harboring the mutations of *TKPR1* and *TKPR2* [40]. *PAL* regulates the formation of flavonoids in *Arabidopsis* and plays a role in scavenging free radicals as well as up-regulating antioxidant enzymes. Moreover, disordered expression of this gene disturbed the metabolism of ROS in plants [68]. At the same time, it has also been reported that *PAL* is also an enzyme that catalyzes the formation of various phenylpropanoid metabolites [69]. Consistent with that, expression of the genes in “phenylpropionic acid biosynthesis/metabolism” pathway has also changed in sterile lines.

In the omics research, gene expression level is often not consistent with its protein accumulation level. A total of 11,324 DEGs and 403 DEPs were identified by omics analysis in the senescence degree of chrysanthemum petals. The author speculates an involvement of transcriptional and epigenetic regulation in petal senescence [70]. RNA-seq and iTRAQ analysis of aphids before and after CMV (cucumber mosaic virus) infection identified 20,550 DEGs, however, only 744 DEPs were obtained [71]. In the study of salt tolerance of Panicum antidotale, 3179 DEGs were detected by RNA-seq at the salt concentration of 100 mM, while only 514 DEPs were identified by Liquid Chromatography Tandem Mass Spectrometer (LC-MS/MS) in the same environment [72]. In the conjoint analysis of the transcriptomic and proteomic data in this study, gene expression level is often not consistent with its protein accumulation level. There are many possible reasons for the above-mentioned phenomenon: first, a transcript may not encode a protein; second, proteins can be transported from other tissues or organs; third, gene transcription may not synchronize with protein translation; fourth, the accumulation of protein is also impacted by degradation; and fifth, protein may be modified after translation [73,74,75,76,77,78]. Consistent with what is mentioned above, several studies have found that protein levels are significantly later than mRNA levels in the time required to reach the same peak [79]. Although there are some differences between the proteome data and the transcriptome data, there is no significant difference in the enrichment pathway, which indicated although significant differences in the number and abundance of differentially expressed genes and proteins but a high level of consistency in expression patterns and metabolic pathways. In addition, the common characteristics of the proteomic data and the qRT-PCR analysis supported the reproducibility and reliability of the transcriptomic data.

Taking this together, the male sterility of celery W99A is high likely to be caused by the changes of “sporopollenin biosynthesis process”, “starch and sucrose metabolism” and “glycolysis/gluconeogenesis”-related process, and genes, including *EVM0000599*(*ALDH3H1*), *EVM0014167*(*ALDH2B4*), *EVM0031399*(*TKPR1*) and *EVM0041100*(*PAL*), may play critical roles in the above-mentioned processes and involve in the determination of the male sterility.

## 4. Materials and Methods

### 4.1. Plant Materials

The celery CMS line W99A and its maintainer line W99B with the same nuclear background were used in this study. Plants were field grown without nutrient and moisture stress at the China Agricultural University, Beijing, China, in 2017. For RNA sequencing, proteome analysis and gene expression analysis, flower buds were collected at the critical stage of pollen abortion (tetrad stages), frozen in liquid nitrogen immediately, and then stored at −80 ℃. W99A corresponds to T01, T02 and T03, and W99B corresponds to T04, T05 and T06. For real-time PCR analysis, three biological and three technical replicates were conducted.

### 4.2. Transcriptomic Analysis

Total RNA from different samples was extracted using TRIzol reagent (Invitrogen, Waltham, MA, USA) according to the manufacturer’s instructions. The purity and integrity of the extracted RNA were detected by Nanodrop and agilent bioanalyzer 2100 system (agilent technologies, Santa Clara, CA, USA), respectively. Sequencing library preparation was done using the NEB Next Ultra^TM^ RNA Library Prep Kit (NEB, Ipswich, MA, USA). After cluster generation, the DNA libraries were sequenced on the Illumina Hiseq platform, and 150 bp paired-end reads were generated.

The RNA-Seq raw sequence data are deposited in the Short Read Archive (SRA) of National Centre for Biotechnology (NCBI) and are available under the accession: PRJNA884666.

### 4.3. Proteomic Analysis

In this study, the isobaric tags for relative and absolute quantification (iTRAQ) technology were chosen for proteomic analysis. Protein extraction was performed using the TCA-acetone procedure [80] and the protein concentration was measured using the Bradford method. The subsequent trypsin digestion, peptide isotope labeling, HPLC fractionation and LC-MS/MS analysis refer to the methods of Zhi et al. [81].

The mass spectrometry proteomics data have been deposited to the ProteomeXchange Consortium via the PRIDE partner repository with the dataset identifier PXD012762. The reviewer account: Username: reviewer_pxd037119@ebi.ac.uk; Password: s8sZB9Tj.

### 4.4. Bioinformatics Analysis

The subsequent differential gene/protein expression analysis and its GO annotation and KEGG enrichment analysis refer to the method of Wang et al. and Young et al. [82], and the celery genome reference to the genome assembled in our lab [83].

### 4.5. Quantitative Real-Time PCR Validation

The extraction and reverse transcription of RNA from flower buds were consistent with those of Cheng et al. [1]. The internal reference base used is celery actin (Gene ID:101260631). The PCR was performed, and the amplification and dissociation curves of ten primers were obtained using an ABI-7500 fluorescence quantitative PCR instrument (Applied Biosystems, Foster City, CA, USA). The primers were designed using primer5 (http://www.premierbiosoft.com/primerdesign/, accessed on 12 May 2021). The data were collected, and the results were analyzed using SPSS software.

### 4.6. PRM Verification of DEPs

PRM is a data acquisition technology based on high-resolution and high-precision mass spectrometry, which can be used to study target protein molecules in targeted quantitative proteomics and is often used to verify proteomic results [84]. Specific experimental methods were carried out in reference to the literature [85].

### 4.7. Biochemical Assay of Male Sterile Lines W99A and Maintainer Line W99B in Celery

Free proline, malondialdehyde (MDA) and hydrogen peroxide contents, SOD, CAT and POD activities and superoxide anion production rate were determined as described in Guo et al. [86]. Three biological replicates were set up for each treatment, and three data were taken for each replicate. Significance was analyzed with Excel 2010 and plotted with Origin 8.0.

## 5. Conclusions

In the present study, transcriptomic and proteomic analyses of the anthers were conducted between the CMS line W99A and its maintainer line W99B at the tetrad stages. In total, 1255 DEGs and 89 DEPs were identified at the transcriptomic and proteomic levels, respectively. Among them, 25 genes were common to both groups. Omics analysis showed that the differential genes/proteins were mainly concentrated in the processes of pollen wall synthesis, cell wall material metabolism, mitochondrial energy metabolism, redox activity, and most of the genes enriched in these pathways were down-regulated in male sterile lines. It can be presumed that the difference is caused by the different expression patterns of mRNA and protein in the same period. The expression of four genes that were closely related to pollen development was significantly decreased in male sterile lines. It is speculated that these genes may lead to plant sterility by inhibiting sporin synthesis, pollen exine synthesis and oxidoreductase activity. Meanwhile, the content and enzyme activity of substances related to redox enzyme activity and redox process in celery male sterile line W99A were measured, and the results showed that they were abnormal in some degree compared with maintainer lines. Based on this, we speculated that genes involved in regulating these pathways are abnormal, resulting in cytoplasmic male sterility. Therefore, this study laid the foundation for future investigation of gene function related to pollen development and cytoplasmic male sterility in celery.

## Data Availability

The RNA-Seq raw sequence data are deposited in the Short Read Archive (SRA) of National Centre for Biotechnology (NCBI) and are available under the accession: PRJNA884666 (https://www.ncbi.nlm.nih.gov/bioproject/PRJNA884666) (accessed on 27 September 2022). The mass spectrometry proteomics data have been deposited to the ProteomeXchange Consortium via the PRIDE partner repository with the dataset identifier PXD012762. The reviewer account: Username: reviewer_pxd037119@ebi.ac.uk; Password: s8sZB9Tj (https://www.ebi.ac.uk/pride/) (accessed on 30 September 2022).

## Supplementary Materials

The following supporting information can be downloaded at: https://www.mdpi.com/article/10.3390/ijms24044194/s1.
