# Supplementary material for: Transcriptomic and Proteomic Analyses of Celery Cytoplasmic Male Sterile Line and Its Maintainer Line
Source: Int J Mol Sci. 2023 Feb 20;24(4):4194. doi: 10.3390/ijms24044194 (PMC9967367; doi:10.3390/ijms24044194)
Supplement: Supplementary file 1 [file ijms-24-04194-s001.zip › Supplementary.pdf]

Supplementary materials

Table S1 Summary of RNA sequencing and assembly

Samples	Total reads	Clean reads	Mapped reads（%）	Unique genes（%）
T01	46,131,424	23,065,712	38,882,611 (84.29%)	36,146,434 (78.36%)
T02	47,762,330	23,881,165	40,137,395 (84.04%)	37,252,942 (78.00%)
T03	51,021,184	25,510,592	42,805,351 (83.90%)	39,789,111 (77.99%)
T04	47,468,316	23,734,158	39,978,670 (84.22%)	37,027,177 (78.00%)
T05	47,172,798	23,586,399	39,763,260 (84.29%)	36,598,988 (77.58%)
T06	57,251,824	28,625,912	48,833,251 (85.30%)	44,905,793 (78.44%)

Table S5 List of DEGs associated with anther and pollen development in celery

Gene ID	Description	Homologous	Regulation(B/A)
EVM0031399	Tetraketide alpha-pyrone reductase 1	LOC108217645	Up
EVM0026892	Protein STRICTOSIDINE SYNTHASE-LIKE 13	LOC108198869	Up
EVM0001985	4-coumarate--CoA ligase-like 1	LOC108198861	Up
EVM0000845	Type III polyketide synthase A	LOC108192424	Up
EVM0009695	Arogenate dehydratase/prephenate dehydratase 6, chloroplastic	LOC108210565	Up
EVM0041100	Phenylalanine ammonia-lyase-like	LOC108224761	Up
EVM0000923	3-oxoacyl-[acyl-carrier-protein] synthase I, chloroplastic-like	LOC108210280	Up
EVM0013046	MFP1 attachment factor 1-like	LOC108217673	Down
EVM0041577	Peroxisome biogenesis factor 10-like	LOC108199858	Down
EVM0036709	Linoleate 13S-lipoxygenase 2-1, chloroplastic-like	LOC108198193	Down

Table S13 Potential regulatory genes involved in each pathway

Gene ID	Description	Homologous (*Arabidopsis*)	Pathway
EVM0031399	Tetraketide α-pyrone reductase 1	TKPR1	sporopollenin biosynthesis process / oxidoreductase activity
EVM0001985	4-coumarate--CoA ligase-like 1	4CL	sporopollenin biosynthesis process
EVM0000845	Type III polyketide synthase A	PKS	sporopollenin biosynthesis process.
EVM0041100	Phenylalanine ammonia-lyase-like	PAL	oxidoreductase activity
EVM0014167	aldehyde dehydrogenase family 2 member B4	ALDH2B4	glycolysis/glycogenesis.
EVM0000599	aldehyde dehydrogenase family 3 member H1	ALDH3H1	glycolysis/glycogenesis
