# Supplementary figures and images for: Transcriptomic and Proteomic Analyses of Celery Cytoplasmic Male Sterile Line and Its Maintainer Line
Source: Int J Mol Sci. 2023 Feb 20;24(4):4194. doi: 10.3390/ijms24044194 (PMC9967367; doi:10.3390/ijms24044194)

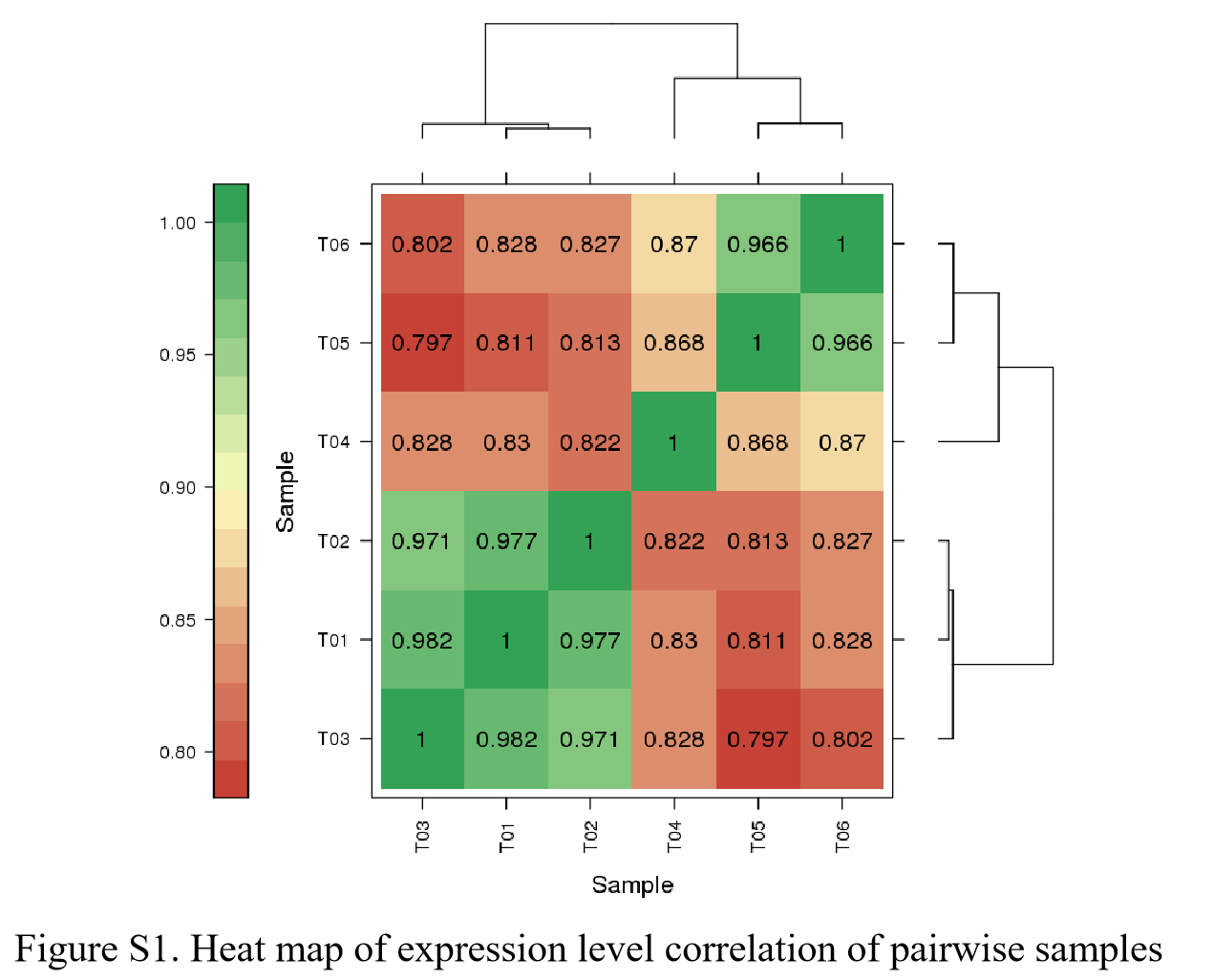

Supplement: Supplementary file 1 [file ijms-24-04194-s001.zip › Figure S1.png]

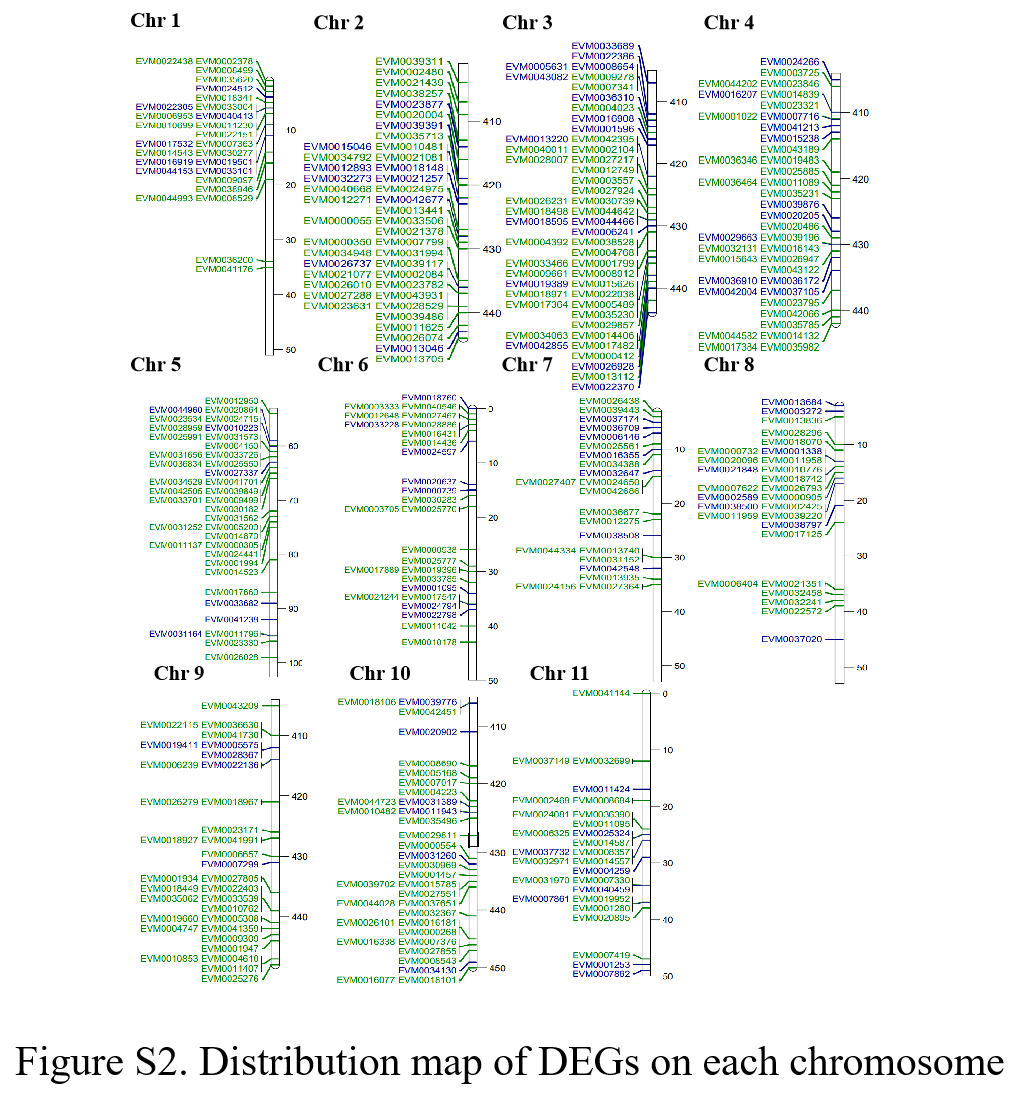

Supplement: Supplementary file 1 [file ijms-24-04194-s001.zip › Figure S2.png]

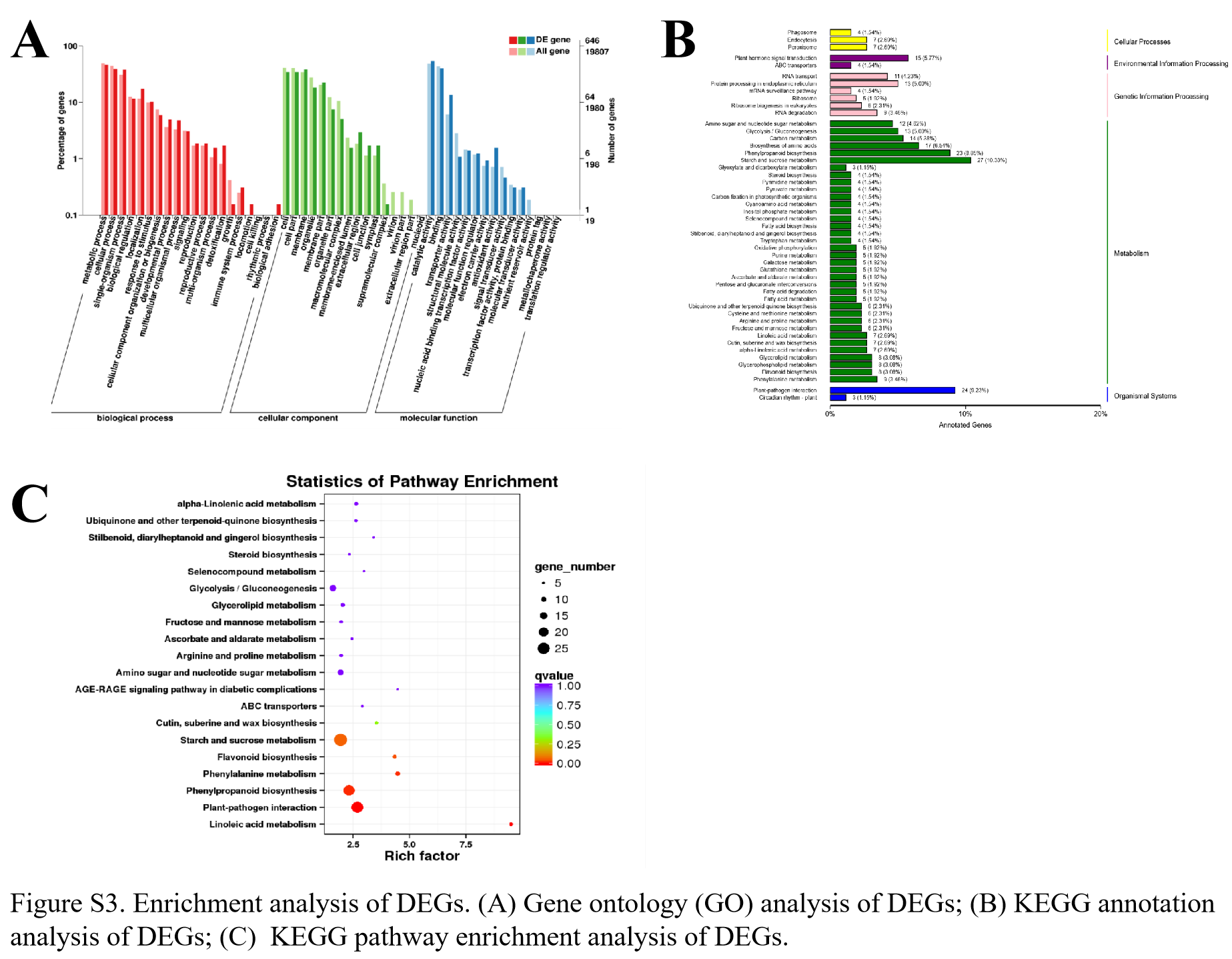

Supplement: Supplementary file 1 [file ijms-24-04194-s001.zip › Figure S3.png]

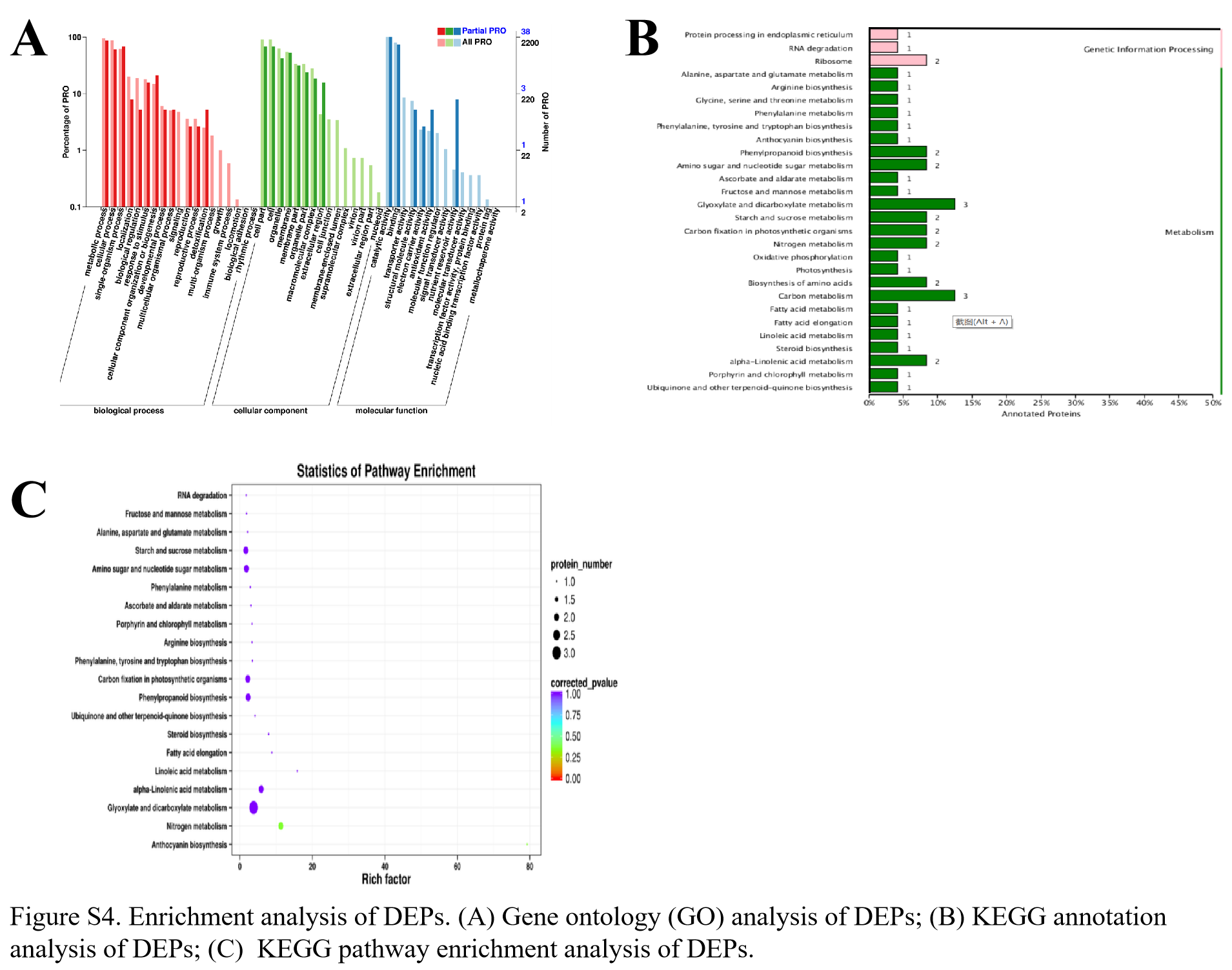

Supplement: Supplementary file 1 [file ijms-24-04194-s001.zip › Figure S4.png]

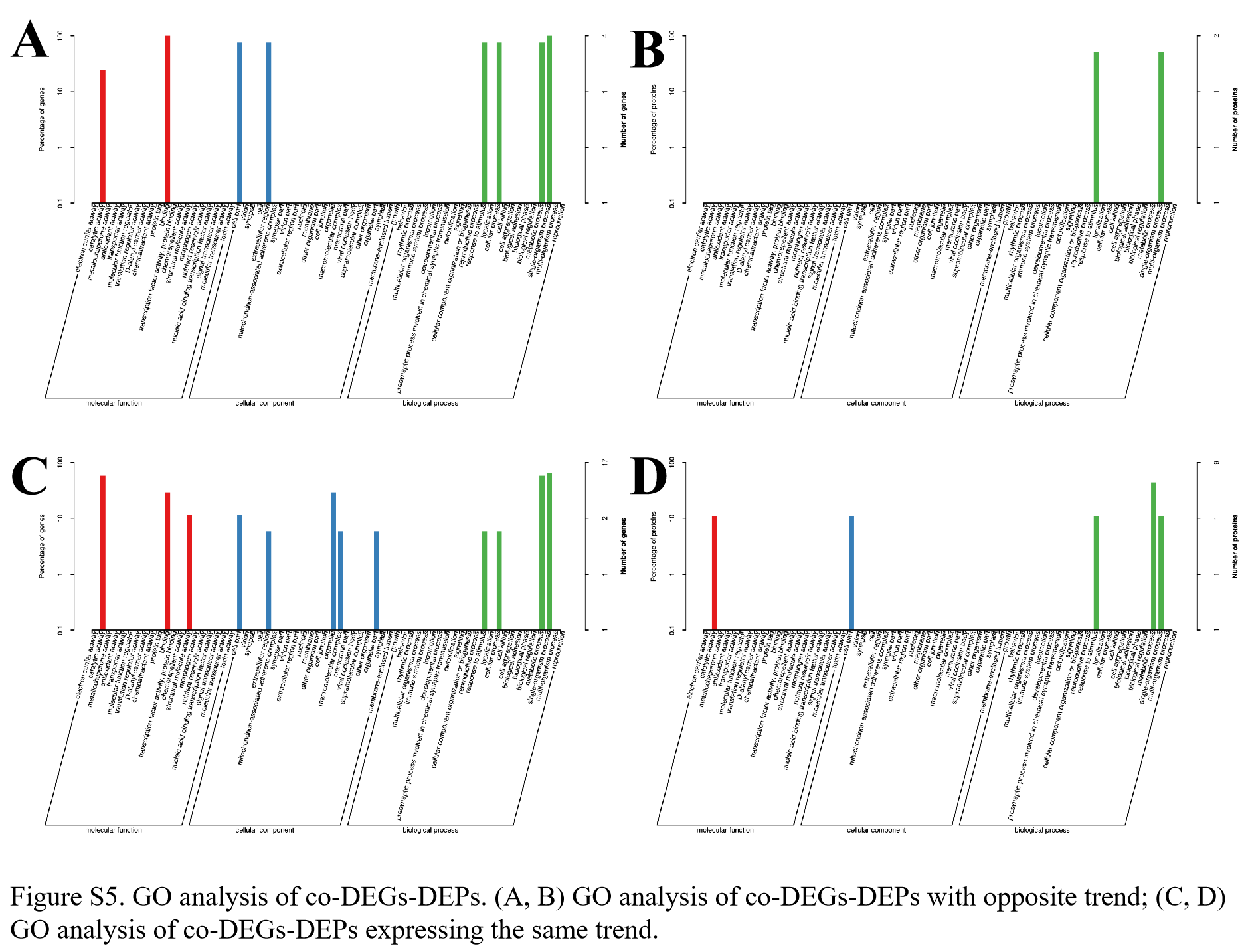

Supplement: Supplementary file 1 [file ijms-24-04194-s001.zip › Figure S5.png]

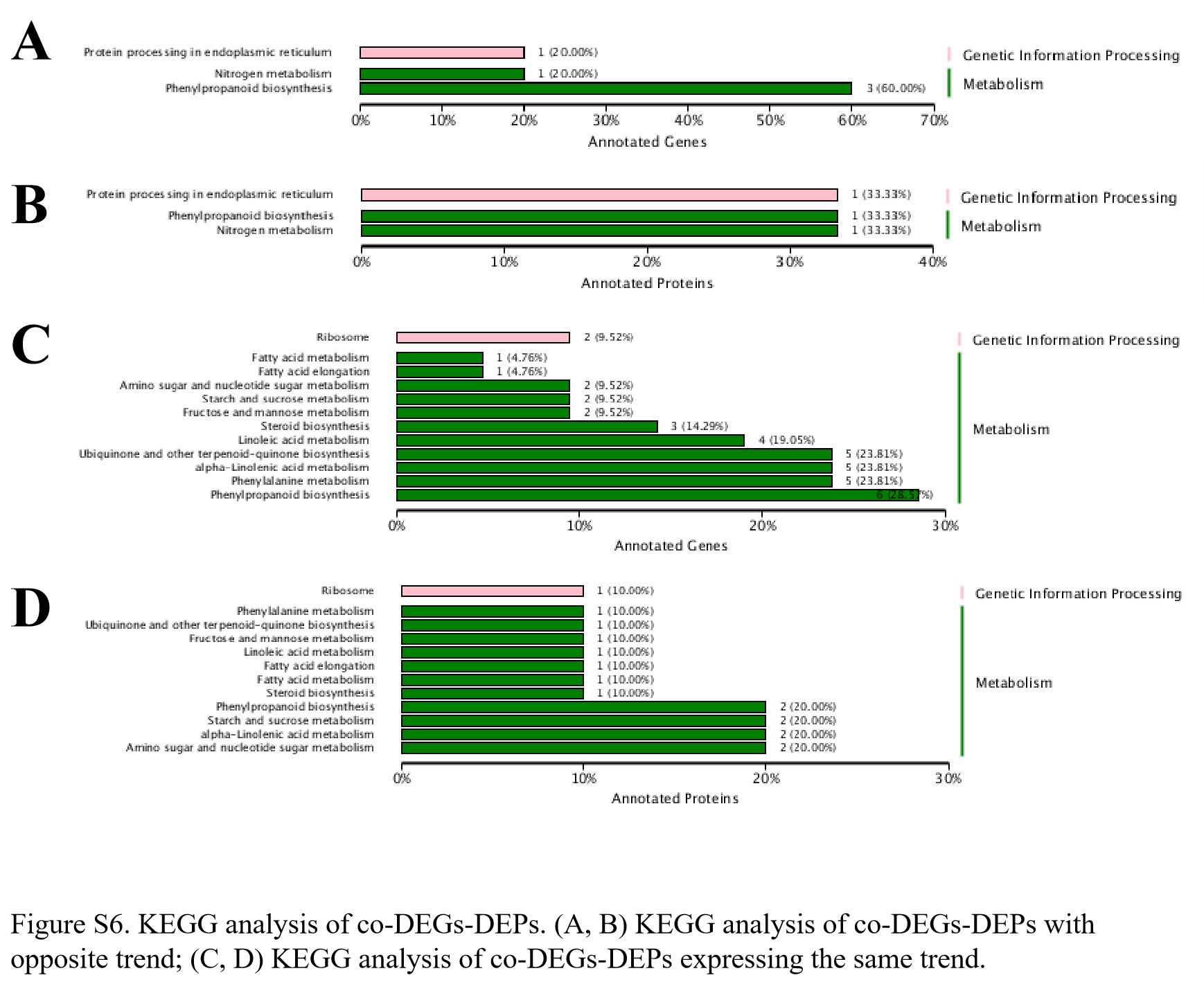

Supplement: Supplementary file 1 [file ijms-24-04194-s001.zip › Figure S6.png]
